# Gestational ages–specific blood pressure patterns and risk of adverse pregnancy outcomes in women with chronic hypertension

**DOI:** 10.1038/s41440-026-02710-9

**Published:** 2026-06-16

**Authors:** Munekage Yamaguchi, Jun Morinaga, Akihito Sagara, Yoshinori Yamanouchi, Azusa Miyashita, Eiji Kondoh

**Affiliations:** 1 Department of Obstetrics and Gynecology, Faculty of Life Sciences, Kumamoto University, Kumamoto, Japan; 2 Department of Clinical Investigation, Kumamoto University Hospital, Kumamoto, Japan

**Keywords:** Blood Pressure, Chronic Hypertension, Gestational Age, Pregnancy Complications, Risk Assessment

## Abstract

Management of chronic hypertension in pregnancy remains uncertain, and current guidelines do not address whether the prognostic significance of blood pressure (BP) varies across gestation. To evaluate gestational age–specific associations between maternal BP in the first half of pregnancy and adverse maternal and neonatal outcomes. We conducted a multicenter registry-based cohort study from April 2022 to March 2023 at 65 tertiary referral centers in Japan. A total of 273 women with chronic hypertension and singleton pregnancies were enrolled before 14 weeks’ gestation (median age, 37 years; IQR, 34–40). Systolic and diastolic BP were assessed at three gestational windows (8–9, 10–13, and 14–18 weeks). Aspirin exposure was treated as time-dependent. The primary outcome was a composite of adverse maternal and neonatal events. Cox proportional hazards models and restricted cubic spline analyses were used. Adverse outcomes occurred in 32.6% (89/273). BP–risk associations differed by timing. No association was observed at 8–9 weeks. At 10–13 weeks, risk increased progressively with higher systolic BP, including excess risk in the moderate range (120–134 mmHg) and the highest risk at ≥135 mmHg (HR, 4.11; 95% CI, 1.14–14.82). At 14–18 weeks, a threshold pattern emerged, with increased risk above 140 mmHg (HR, 2.19; 95% CI, 1.40–3.43). Associations were weaker among women who initiated aspirin before 10 weeks, although interaction was not statistically significant. In chronic hypertension, maternal BP during 10–13 weeks of gestation carries heightened prognostic relevance. These findings support gestational age–specific risk assessment and motivate evaluation of early preventive strategies.

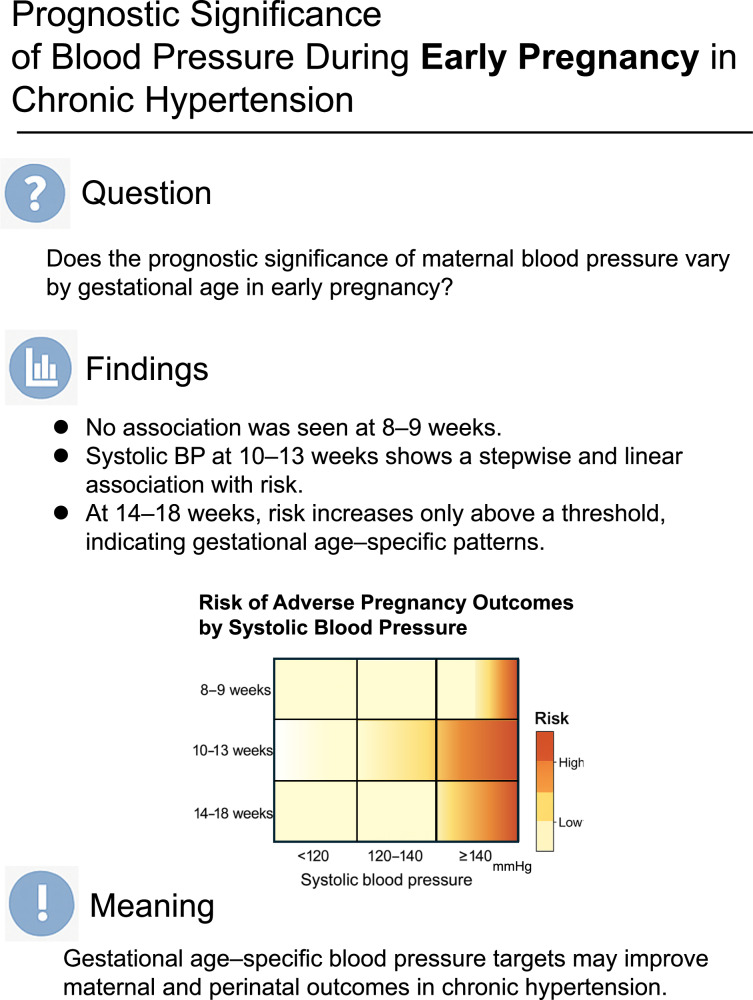

## Introduction

Chronic hypertension, defined as hypertension diagnosed before pregnancy or before 20 weeks of gestation, is one of the major subtypes of hypertensive disorders of pregnancy (HDP). The prevalence of chronic hypertension during pregnancy has doubled from 1.8% to 3.7% over the past 15 years [1]. Chronic hypertension is associated with increased maternal risks, including preeclampsia, severe hypertension, placental abruption, and stroke, as well as fetal and neonatal complications such as medically indicated preterm delivery, small-for-gestational-age (SGA) infants, stillbirth, and neonatal intensive care unit (NICU) admission [2, 3]. Despite these well-established risks, major guidelines—including those from the American College of Obstetricians and Gynecologists (ACOG), the International Society for the Study of Hypertension in Pregnancy (ISSHP), and the Royal College of Obstetricians and Gynecologys (RCOG)—differ in their recommended blood pressure (BP) targets, and few have addressed how these targets should be applied at different gestational stages [4–6]. Most studies and clinical guidelines have treated the first half of pregnancy as a uniform period, without considering gestational age–specific variations in risk. This highlights the need to clarify whether specific time points in early pregnancy are particularly critical in determining the impact of elevated BP on adverse outcomes.

Recent evidence shows that initiating antihypertensive treatment before 23 weeks of gestation in women with mild chronic hypertension reduces major adverse pregnancy outcomes without affecting neonatal birth weight [7]. A secondary analysis further demonstrated that stricter BP control ( < 130/80 mmHg) significantly lowered the incidence of severe preeclampsia and hypertension compared to less stringent targets [8]. However, these studies did not explore whether the timing of BP control during early gestation influences adverse maternal and neonatal outcomes. Our previous retrospective study found that both the level and timing of BP elevation were predictive of early-onset preeclampsia [9]. Notably, elevated systolic BP at 14–15 weeks was more strongly associated with risk than similar levels at 12–13 weeks. These findings suggest that the prognostic relevance of BP depends not only on its absolute value but also on the gestational age at measurement. Since trophoblast invasion and the establishment of maternal–fetal circulation are largely complete by 14 weeks of gestation [10, 11], early BP control may be critical in minimizing adverse pregnancy outcomes.

Therefore, this multicenter registry-based cohort study aimed to evaluate how both the level and timing of BP in the first half of pregnancy are associated with pregnancy outcomes among women with chronic hypertension.

Point of view

- Clinical relevance: The association between maternal blood pressure and adverse pregnancy outcomes differed according to gestational age in early pregnancy, suggesting the potential value of gestational age–specific blood pressure management.
- Future direction: Prospective studies are needed to determine whether gestational age–specific blood pressure targets and earlier preventive interventions can improve maternal and neonatal outcomes.
- Consideration for the Asian population: This multicenter Japanese study suggests that gestational age–specific blood pressure assessment may help optimize the management of chronic hypertension in Asian pregnant women.

## Methods

### Study design

This multicenter, prospective registry-based cohort study was conducted between April 2022 and March 2023 at 65 institutions in Japan, in collaboration with the Maternal–Fetal Intensive Care Unit Liaison Council. The study was designed to evaluate the association between early pregnancy BP and maternal, fetal, and neonatal outcomes in pregnant women with chronic hypertension. The protocol was approved by the ethics committee of Kumamoto University (approval number: 2400), and the study was conducted in accordance with the Declaration of Helsinki. All authors had full access to the data and were responsible for its accuracy and adherence to the protocol.

### Setting and participants

Eligible participants were women with pre-existing or newly diagnosed chronic hypertension and a viable singleton pregnancy before 14 weeks’ gestation (Fig. S1A). Newly diagnosed chronic hypertension was defined as systolic BP ≥ 140 mmHg, diastolic BP ≥ 90 mmHg, or both, recorded before 14 weeks without prior history. Exclusion criteria were secondary or white coat hypertension, multiple gestation, maternal age <20 years, non-Asian ethnicity, severe hypertension at enrollment (systolic ≥160 mmHg or diastolic ≥110 mmHg), pregnancy loss before 20 weeks, or physician judgment of ineligibility. These criteria were applied at the time of enrollment at each participating institution; therefore, individuals meeting these criteria were not registered in the study database.

### Variables

The primary outcome was a composite of maternal and neonatal events (Fig. S1B). Maternal components included superimposed preeclampsia before 34 weeks, severe hypertension after 20 weeks, HDP–related preterm delivery before 34 weeks, HELLP syndrome, placental abruption, eclampsia, stroke, and HDP–related pregnancy loss. Neonatal components included SGA infants, defined as birthweight below the 10th percentile for gestational age and sex, and neonatal intensive care unit (NICU) admission exceeding one week. Definitions followed established criteria (see Supplementary Methods).

### Data sources and measurement

Participants were enrolled before 20 weeks of gestation, with confirmation of chronic hypertension prior to 14 weeks. Written informed consent was obtained from all participants. Some BP measurements during early pregnancy had been recorded as part of routine prenatal care prior to enrollment.

BP values were abstracted from maternity record books and hospital records and represent clinic blood pressure measurements obtained during routine prenatal visits at each participating institution. Home blood pressure measurements were not used in this study. When multiple readings were available, at a single visit, the highest value was used for analysis. Across visits within each gestational window, the maximum recorded BP value was used. Demographic and clinical information, including parity, conception method, prior hypertensive disorders, delivery outcomes, and neonatal data, was obtained from electronic medical records. Data were entered into a secure cloud-based platform (Microsoft 365 SharePoint, Microsoft Corporation, Redmond, WA, USA).

All participants were prospectively followed from enrollment until delivery or termination of pregnancy. Clinical management, including antihypertensive treatment, fetal surveillance, and timing of delivery, was at the discretion of each participating facility.

### Bias

To minimize sampling bias, this study was conducted as a multicenter collaborative effort across 65 institutions in Japan, ensuring that the cohort reflected the characteristics of pregnant women with chronic hypertension nationwide. To reduce information bias, research collaborators photographed relevant pages of participants’ maternity record books, which were centrally uploaded for standardized data extraction. In addition, patient data were recorded in a unified format on a secure cloud-based platform, promoting consistency across institutions.

### Study size

A sample size of 273 was determined by feasibility across 65 centers within the one-year study period. The sample size was determined by the number of eligible women enrolled during the study period across participating institutions. No formal power calculation was performed, given the exploratory nature of this analysis, but all available cases were included to maximize statistical power and generalizability.

### Quantitative variables

BP (systolic and diastolic) was the primary quantitative variable. Values were analyzed as continuous variables, modeled per 5–mmHg increment, and assessed for nonlinearity using restricted cubic splines. For clinical interpretability, BP was also categorized based on both guideline-recommended thresholds (135/85 mmHg, per RCOG guidelines), applied to clinic blood pressure measurements in this study, and spline-derived cutoff points.

### Statistical analysis

Baseline characteristics were summarized using medians with interquartile ranges (IQRs) for continuous variables and proportions for categorical variables. Participants were categorized into three gestational age groups based on the timing of BP measurement: 8–9, 10–13, and 14–18 weeks.

Cox proportional hazards models were used to estimate hazard ratios (HRs) for the composite outcome per 5–mmHg increment in systolic and diastolic BP within each gestational window, accounting for clustering by facility. Multivariable Cox models were adjusted for prespecified covariates: maternal age ( ≥ 37 years), body mass index at enrollment, and HDP history. Aspirin exposure during each interval (8–9, 10–13, and 14–18 weeks) was defined according to initiation before the start of each interval, and antihypertensive drug use was classified into hierarchical categories (details in Supplementary Methods). Restricted cubic spline (RCS) regression was applied to explore potential non-linear associations between BP and outcomes, using 120 mmHg for systolic BP and 80 mmHg for diastolic BP as reference values. Based on the spline curves and clinical relevance, categorical BP groups were defined using both RCOG guideline-recommended target BP (135/85 mmHg) and spline-derived cutoffs. Cox models were then fitted to estimate HRs across categories, and linear trends were tested with Wald statistics.

Subgroup analyses were conducted to assess effect modification by aspirin use. Interaction terms between aspirin use and BP levels were included in Cox models, and interaction effects were evaluated using Wald tests. All analyses were performed using STATA version 19.5 (StataCorp, College Station, TX). A two-sided *p* < 0.05 was considered statistically significant.

## Results

A total of 273 participants were enrolled in this study (Fig. S1B). The clinical characteristics are presented in Table S1. The median maternal age was 37 years (IQR, 34–40), and 46.9% of the participants were nulliparous. Conception via in vitro fertilization and embryo transfer accounted for 29.3% of the pregnancies. The median body mass index at enrollment was 28.5 kg/m² (IQR, 23.6–32.0). Among multiparous women (*n* = 145), 60.7% had a history of HDP. The prevalence of current smoking at enrollment was 4.4%. The median gestational age was 9 weeks (IQR, 8–11) at the initial visit and 14 weeks (IQR, 12–17) at enrollment.

Regarding hypertension status, 30.4% of participants were newly diagnosed during the current pregnancy, while 69.6% had a preexisting diagnosis. At enrollment, nearly half were not receiving any antihypertensive medication, and aspirin was initiated by 14 weeks in 49.1%. Both systolic and diastolic BP declined significantly across gestation, from median values of 142.5/91 mmHg at 8–9 weeks to 134/84 mmHg at 14–18 weeks (Fig. S2, *p* < 0.001 for trend).

Perinatal outcomes are summarized in Table 1. The median gestational age at delivery was 38 weeks (IQR, 37–39). Cesarean delivery was performed in 59.7% (163 of 273). Early-onset preeclampsia occurred in 10.6% (29 of 273), and severe hypertension at or after 20 weeks in 17.9% (49 of 273). HDP-related preterm delivery before 34 weeks was observed in 8.1% (22 of 273). Pregnancy loss related to HDP occurred in 1.5% (4 of 273). The median birth weight was 2814.5 g (IQR, 2440–3124), with 12.1% (33 of 273) SGA and 18.3% (50 of 273) NICU admission for more than one week. Overall, composite adverse outcomes occurred in 32.6% (89 of 273), and the proportion of ongoing pregnancies free from adverse outcomes was 81.9% (95% confidence interval [CI], 76.8–86.0) at 34 weeks and 70.8% (95% CI, 64.9–75.8) at 37 weeks (Fig. S3).

**Table 1:** Perinatal outcomes

Outcomes	*n* (available)	Value, *n* (%) or Median [IQR]
Composite adverse outcome	273	89 (32.6)
**Maternal outcomes**		
Gestational age at delivery, weeks	273	38 [37,39]
Cesarean delivery	273	163 (59.7)
Early-onset superimposed preeclampsia before 34 weeks	273	29 (10.6)
Severe hypertension at ≥ 20 weeks	273	49 (17.9)
HDP-related preterm delivery before 34 weeks	273	22 (8.1)
HELLP syndrome	273	1 (0.4)
Placental abruption	273	2 (0.7)
Eclampsia	273	1 (0.4)
Stroke	273	1 (0.4)
**HDP-related pregnancy loss**		
Termination	273	1 (0.4)
Fetal death	273	3 (1.1)
**Neonatal outcomes**		
Birth weight, g	270	2814.5 [2440, 3124]
Apgar score at 1 min	272	8 [8, 8]
Apgar score at 5 min	272	9 [9, 9]
Umbilical artery pH	270	7.29 [7.25, 7.33]
SGA	273	33 (12.1)
NICU stay > 1 week	273	50 (18.3)

*IQR* interquartile range, *HDP* hypertensive disorders of pregnancy, *HELLP* hemolysis, elevated liver enzymes, low platelet count, *SGA* small for gestational age, *NICU* neonatal intensive care unit

**Fig. 1:**
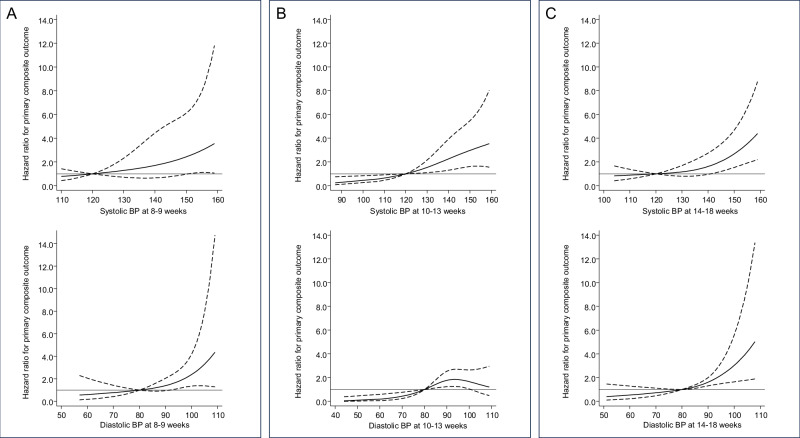
Associations between blood pressure and risk of adverse pregnancy outcome in the first half of pregnancy. Adjusted hazard ratios and 95% confidence intervals for the primary composite outcome are shown based on restricted cubic spline regression models, stratified by gestational age window and blood pressure (BP) type. The solid curve represents the estimated hazard ratio, and the dotted curves represent the 95% confidence intervals. Reference values were set at 120 mmHg for systolic BP and 80 mmHg for diastolic BP, reflecting standard normotensive thresholds. Models were adjusted for maternal characteristics and clinical covariates as described in the Methods, with clustering by facility. **A** BP measured at 8–9 weeks: Risk markedly increased at diastolic BP > 91 mmHg and systolic BP > 150 mmHg. **B** BP at 10–13 weeks: Risk increased consistently with rising systolic BP, while lower diastolic BP ( < 80 mmHg) was associated with significantly reduced risk. Diastolic BP between 80 and 100 mmHg showed persistently elevated risk, although estimates above 100 mmHg were not statistically significant, possibly due to limited sample size. **C** BP at 14–18 weeks: Systolic BP > 141 mmHg and diastolic BP > 80 mmHg were both significantly associated with increased risk

To assess the association between BP and composite adverse outcomes, the first half of pregnancy period was categorized into three intervals: 8–9, 10–13, and 14–18 weeks. Cox proportional hazards models showed that higher systolic BP and diastolic BP were consistently associated with increased risk across all intervals (Table 2). To explore optimal BP thresholds for predicting adverse outcomes, RCS analyses were performed, using systolic BP 120 mmHg and diastolic BP 80 mmHg as normotensive reference values. RCS analyses identified gestational age–dependent patterns of risk (Fig. 1). At 8–9 weeks, elevated risk emerged only at very high BP levels (Fig. 1A; systolic >150 mmHg, diastolic >91 mmHg). At 10–13 weeks, systolic BP showed a graded association across the entire range, with progressively lower risk at lower values. For diastolic BP, risk increased above 80 mmHg, whereas risk decreased progressively with values below 80 mmHg (Fig. 1B). By 14–18 weeks, a clear threshold effect was observed, with systolic BP > 141 mmHg and diastolic BP > 80 mmHg associated with elevated risk (Fig. 1C). Thus, BP–risk associations were predominantly linear at 10–13 weeks, whereas a threshold-type relationship emerged at 14–18 weeks.

**Table 2:** Gestational age–specific associations between blood pressure and risk of adverse pregnancy outcomes

	No. of events/ No. at risk	HR	95% CI	*P*
Maximum systolic BP (per 5 mmHg)				
8–9 weeks	39/117	1.22	(1.06, 1.40)	0.007
10–13 weeks	69/221	1.18	(1.08, 1.29)	≤0.001
14–18 weeks	82/257	1.20	(1.08, 1.32)	0.001
Maximum diastolic BP (per 5 mmHg)				
8–9 weeks	45/131	1.30	(1.11, 1.51)	0.005
10–13 weeks	79/250	1.14	(1.04, 1.26)	0.007
14–18 weeks	84/264	1.29	(1.14, 1.45)	<0.001

Cox proportional hazards models were adjusted for maternal characteristics and clinical covariates as described in the Methods. Models also accounted for clustering by medical facility. Participants with systolic BP ≥ 160 mmHg or diastolic BP ≥ 110 mmHg were excluded from analyses within each gestational time window

*HR* hazard ratio, *CI* confidence interval, *BP* blood pressure

To clarify the clinical significance of RCS-identified thresholds, we integrated them with RCOG-recommended cutoffs. As both Cox models and RCS analyses showed stronger associations from 10 weeks onward, risk stratification was performed for the 10–13 and 14–18 week intervals. Systolic BP showed a graded association with outcomes in both periods (Table 3), with the highest risk at ≥135 mmHg during 10–13 weeks (HR 4.11; 95% CI, 1.14–14.82) and somewhat lower but still significant risk at ≥140 mmHg during 14–18 weeks (HR 2.19; 95% CI, 1.40–3.43). Notably, even the moderate-risk group (120–134 mmHg) at 10–13 weeks showed a trend toward increased risk (HR 2.69; 95% CI, 0.73–9.89). Diastolic BP ≥ 85 mmHg was consistently associated with increased risk at both intervals (HR 2.32 [95% CI, 1.28–4.20] and 2.05 [95% CI, 1.26–3.34], respectively), whereas the moderate-risk group ( > 80 to <85 mmHg) carried no significant excess risk. These stratifications confirmed a stepwise rise in risk (*P* < 0.05 for trend in each period), supporting the utility of both spline-derived and guideline-based BP thresholds.

**Table 3:** Gestational age–specific risk of adverse pregnancy outcomes by blood pressure categories

	No. of events/ No. at risk	HR	95% CI	*P*	*P* for trend
Maximum systolic BP					
10–13 weeks					
Low risk group ( ≤ 120 mmHg)	3/20	1.00	-	-	0.020
Moderate risk group ( > 120 to < 135 mmHg)	18/69	2.69	(0.73, 9.89)	0.136	
High risk group ( ≥ 135 mmHg)	48/132	4.11	(1.14, 14.82)	0.031	
14–18 weeks					
Low risk group ( ≤ 135 mmHg)	39/143	1.00	-	-	0.001
Moderate risk group ( > 135 to < 140 mmHg)	10/37	1.18	(0.64, 2.19)	0.593	
High risk group ( ≥ 140 mmHg)	33/77	2.26	(1.46, 3.54)	<0.001	
Maximum diastolic BP					
10–13 weeks					
Low risk group ( ≤ 80 mmHg)	10/50	1.00	-	-	0.012
Moderate risk group ( > 80 to <85 mmHg)	8/31	1.26	(0.52, 3.04)	0.611	
High risk group ( ≥ 85 mmHg)	61/169	2.32	(1.28, 4.20)	0.006	
14–18 weeks					
Low risk group ( ≤ 80 mmHg)	18/79	1.00	-	-	0.013
Moderate risk group ( > 80 to <85 mmHg)	17/55	1.43	(0.78, 2.65)	0.248	
High risk group ( ≥ 85 mmHg)	48/129	2.05	(1.26, 3.34)	0.004	

Cox proportional hazards models were adjusted for maternal characteristics and clinical covariates as described in the Methods, with clustering by medical facility. Participants with systolic BP ≥ 160 mmHg or diastolic BP ≥ 110 mmHg were excluded from each gestational-phase-specific analysis. *HR* hazard ratio, *CI* confidence interval, *BP* blood pressure

To assess effect modification by aspirin, subgroup analyses were conducted using Cox models stratified by aspirin exposure, focusing on systolic BP given its consistent associations with adverse outcomes (Table 3). As shown in Table 4, during both the 8–9 and 10–13 week intervals, elevated systolic BP was significantly associated with an increased risk of adverse outcomes in participants who were not receiving aspirin (HR 1.27, *P* = 0.007 at 8–9 weeks; HR 1.22, *P* = 0.002 at 10–13 weeks). In contrast, among those who received aspirin, the associations were weaker and not statistically significant (HR 1.01, *P* = 0.940 at 8–9 weeks; HR 1.09, *P* = 0.246 at 10–13 weeks). Although formal interaction tests did not reach statistical significance (*P* for interaction = 0.265 and 0.275, respectively), the direction and magnitude of these associations are compatible with a possible modifying effect of aspirin. By 14–18 weeks, elevated systolic BP was associated with increased risk regardless of aspirin use, with similar effect sizes in both groups (HR 1.18 and 1.21, respectively), suggesting that the mitigating effect of aspirin on systolic BP-related risk may be limited to earlier gestational periods.

**Table 4:** Maximum Systolic Blood Pressure and Risk of Adverse Pregnancy Outcomes Across Aspirin and Gestational Age Subgroups

Gestational age interval	Aspirin use	No. of events / No. at risk	HR (per 5 mmHg systolic BP)	95% CI	*P*	*P* for interaction
8–9 weeks	No	34/106	1.27	(1.07, 1.51)	0.007	0.265
	Yes	5/11	1.01	(0.73, 1.40)	0.940	
10–13 weeks	No	53/182	1.22	(1.07, 1.38)	0.002	0.275
	Yes	16/39	1.09	(0.94, 1.25)	0.246	
14–18 weeks	No	43/128	1.19	(1.04, 1.36)	0.004	0.869
	Yes	39/129	1.20	(1.04, 1.40)	0.013	

Cox proportional hazards models were adjusted for maternal characteristics and clinical covariates as described in the Methods. Models also accounted for clustering by medical facility. Participants with systolic BP ≥ 160 mmHg or diastolic BP ≥ 110 mmHg were excluded from analyses within each gestational time window

*HR* hazard ratio, *CI* confidence interval, *BP* blood pressure

## Discussion

Our study demonstrates that in women with chronic hypertension, both the magnitude and the timing of BP elevation in the first half of pregnancy are independently associated with adverse perinatal outcomes. Specifically, 10–13 weeks of gestation emerged as a critical window during which even modest systolic BP elevations (120–134 mmHg) conferred increased risk, whereas BP before 10 weeks showed no association and after 14 weeks exhibited a threshold effect. Among the components of the composite outcome, the most frequent events were NICU stay > 1 week, severe hypertension at ≥ 20 weeks, and SGA, followed by early-onset superimposed preeclampsia and HDP-related preterm delivery before 34 weeks, whereas severe maternal complications such as eclampsia or stroke were rare.

### Biological implications of gestational age–specific BP patterns

Malplacentation is a well-recognized contributor to preeclampsia and fetal growth restriction [10, 11]. Previous clinical trials, including the CHAP trial, have shown that initiating antihypertensive therapy for women with mild chronic hypertension before 23 weeks can improve outcomes without impairing fetal growth [7]. However, these studies did not examine whether the prognostic significance of BP varies across different gestational windows. In our study, Cox proportional hazards models showed that both systolic and diastolic BP were significantly associated with adverse outcomes at each gestational window examined. RCS analyses further suggested a gestational age–dependent pattern: BP before 10 weeks showed no clear association with adverse outcomes, potentially reflecting limited maternal perfusion of the placenta during this stage. Between 10 and 13 weeks, higher systolic BP was associated with a progressively greater risk, a period that largely overlaps with spiral artery remodeling and the development of maternal–fetal circulation [10, 11]. After 14 weeks, the association appeared more threshold-like, suggesting reduced placental sensitivity once its vascular architecture is largely established. Together, these findings support the hypothesis that there may be a biologically sensitive period in early placental development during which maternal hemodynamics exert heightened influence on pregnancy outcomes.

### Implications for clinical practice

Clinical management of chronic hypertension in pregnancy remains cautious—particularly in the first trimester—due to concerns that excessive BP reduction could impair placental perfusion and restrict fetal growth. Current guidelines differ in their recommended thresholds and targets: ACOG advises initiating or adjusting therapy at 140/90 mmHg without specifying a target; ISSHP recommends a target diastolic BP of 85 mmHg; and RCOG advises aiming for 135/85 mmHg [4–6]. None of these guidelines incorporate gestational age–specific thresholds. Our findings suggest that even “mildly elevated” systolic BP (120–134 mmHg) at 10–13 weeks may be associated with increased risk, as these values exceed typical early-pregnancy levels in normotensive women (100–120 mmHg) [12]. In Table 4, the association between elevated blood pressure and adverse outcomes appeared attenuated among women who initiated aspirin before 10 weeks, suggesting a potential risk reduction associated with early aspirin use. However, formal interaction analyses did not show statistically significant effect modification by aspirin use. The sample size in this subgroup was limited, and these findings should be interpreted with caution. While the role of aspirin in chronic hypertension remains uncertain [13], earlier initiation of aspirin prior to 10 weeks may coincide with biologically sensitive windows of placental development (10–13 weeks), when maternal hemodynamics exert heightened influence on pregnancy outcomes. These observations generate the hypothesis that earlier aspirin initiation, in combination with BP control, warrants further evaluation in prospective studies.

## Limitations

This study has limitations. Although prospectively registered, this study was observational, and management decisions were not standardized. Antihypertensive drug selection, treatment initiation or adjustment, and BP targets were determined at the discretion of treating physicians, which may have influenced both BP levels and clinical outcomes. BP measurements were obtained as part of routine clinical practice across participating institutions and were not standardized across centers, which may have introduced measurement variability. Although measurement frequency was generally similar across patients, minor differences in measurement intensity cannot be excluded. Residual confounding from unmeasured factors (e.g., provider practice patterns, socioeconomic status) is possible. The study population was derived from tertiary referral centers in Japan, which may also limit the generalizability of our findings to the broader population of pregnancies complicated by hypertension. Another limitation is that home blood pressure measurements were not collected in this registry; therefore, our findings are based solely on blood pressure measurements obtained in clinical settings. Larger, multiethnic prospective studies and randomized trials testing gestational age–specific interventions are needed to confirm and extend our findings.

### Perspective of asia

Chronic hypertension during pregnancy is becoming an increasingly important clinical issue in Asian countries because of advancing maternal age. In this multicenter Japanese cohort, we found that the association between maternal blood pressure and adverse pregnancy outcomes differed according to gestational age in early pregnancy. Blood pressure at 8–9 weeks showed no clear association with adverse outcomes, suggesting that this period may still represent a potentially modifiable stage for improving pregnancy outcomes. During 10–13 weeks, blood pressure showed a predominantly linear association with adverse outcomes, whereas a threshold-type association was observed after 14 weeks of gestation. These findings suggest that management strategies for chronic hypertension in pregnancy may need to be adapted according to gestational age rather than applying a uniform blood pressure target throughout early pregnancy. Further prospective studies across diverse populations, including Asian populations, are warranted to establish gestational age–specific management strategies.

## Conclusion

The prognostic significance of maternal blood pressure in women with chronic hypertension is not uniform across the first half of pregnancy. Our findings support a paradigm shift from uniform management of chronic hypertension in pregnancy toward gestational age–specific risk assessment and intervention, particularly during the 10–13 week window. Implementing stage-specific BP targets and early preventive strategies may improve outcomes in this high-risk population.

## Supplementary information

Supplementary Information

htr-2025-1102-File009
