# Supplementary material for: Gestational ages–specific blood pressure patterns and risk of adverse pregnancy outcomes in women with chronic hypertension
Source: Hypertens Res. 2026 Jun 16;49(8):2377–85. doi: 10.1038/s41440-026-02710-9 (PMC13437243; doi:10.1038/s41440-026-02710-9)
Supplement: Supplementary file 1 [file 41440_2026_2710_MOESM1_ESM.docx]

**Supplemental Material**

**Supplementary Methods**

***Detailed Outcome Definitions***

Severe hypertension was defined as systolic blood pressure (BP) ≥160 mmHg or diastolic BP ≥110 mmHg. HELLP syndrome was diagnosed based on the Tennessee classification ^[1]^. Hypertensive disorders of pregnancy (HDP)-related pregnancy loss included both medically indicated termination and intrauterine fetal death. Superimposed preeclampsia was defined as chronic hypertension diagnosed before 20 weeks of gestation, complicated after 20 weeks by new-onset proteinuria or maternal organ dysfunction—including renal, hepatic, neurologic, hematologic, or placental involvement (e.g., fetal growth restriction)—in the absence of other underlying systemic conditions. Small for gestational age was defined as birthweight <10th percentile for gestational age and sex ^[2]^.

***Gestational Age Windows***

The gestational age windows (8–9, 10–13, and 14–18 weeks) were selected to align with key stages of placental development: early trophoblast invasion (8–9 weeks), establishment of maternal–fetal circulation (10–13 weeks), and onset of mature placental perfusion (14–18 weeks).

***Longitudinal BP Trends***

To evaluate longitudinal changes in BP during the first half of pregnancy, mixed-effects models ^[3]^ were constructed with subject ID as a random intercept to estimate BP trajectories from the first prenatal visit through 19 weeks of gestation. Robust standard errors ^[4]^ were applied, and time trends were assessed using ANOVA.

***Kaplan–Meier Curves***

Kaplan–Meier curves were constructed to illustrate the proportion of ongoing pregnancies without composite outcomes across gestation.

***Body Mass Index Categories***

BMI at enrollment was categorized as <18.5, 18.5–24.9, 25–29.9, 30–34.9, and ≥35.

***Facility-Level Clustering***

Robust standard errors were calculated using a sandwich estimator with facility-level clustering (STATA option: vce(cluster facility)).

***Classification of Antihypertensive Medication Use***

At enrollment (before 14 weeks of gestation), antihypertensive medication use was classified into five mutually exclusive categories: no medication, calcium channel blocker, labetalol, methyldopa, or others.

For women prescribed multiple agents concurrently, a hierarchical decision rule was applied to ensure unique assignment:

1. Calcium channel blocker (if used, irrespective of other drugs)
2. Labetalol (if used, without calcium channel blocker)
3. Methyldopa (if used, without calcium channel blocker or labetalol)
4. Others (if agents other than the above were used exclusively)
5. No medication (if no antihypertensives were prescribed)

This hierarchical classification allowed for consistent grouping of participants and avoided overlap in exposure categories.

***Definition of Aspirin Use During Each Interval***

Aspirin exposure was treated as a time-dependent variable and defined separately for each gestational interval (8–9, 10–13, and 14–18 weeks).

For a given interval, aspirin use was coded as “**yes**” if aspirin had been initiated on or before the first week of that interval. Conversely, aspirin use was coded as “**no**” if aspirin was initiated after the first week of the interval or if no aspirin was prescribed at all.

For example:

・During the 8–9 week interval, aspirin use was coded as “yes” if initiated at 8 weeks or earlier, and “no” if initiated at ≥9 weeks or not prescribed.

・During the 10–13 week interval, aspirin use was coded as “yes” if initiated at ≤10 weeks, and “no” if initiated after 10 weeks or not prescribed.

・The same logic was applied to the 14–18 week interval.

This operational definition ensured that aspirin exposure status was aligned with biologically relevant gestational timing for each analytic window.

**Supplementary References**

1. Martin JN Jr, Rinehart BK, May WL, Magann EF, Terrone DA, Blake PG. The spectrum of severe preeclampsia: comparative analysis by HELLP (hemolysis, elevated liver enzyme levels, and low platelet count) syndrome classification. Am J Obstet Gynecol. 1999;180:1373-1384.
2. Itabashi K, Miura F, Uehara R, Nakamura Y. New Japanese neonatal anthropometric charts for gestational age at birth. Pediatr Int. 2014;56:702-708.
3. Laird, N. M., & Ware, J. H. Random effects models for longitudinal data. Biometrics. 1982;38:963-974.
4. White H. A heteroskedasticity-consistent covariance matrix estimator and a direct test for heteroskedasticity. Econometrica. 1980;48:817–838.

**Supplementary Tables**

***Table S1. Clinical characteristics of study participants***

| Clinical characteristics | n (available) | Value, n (%)  or Median [IQR] |
| --- | --- | --- |
| Clinical demographics |  |  |
| Maternal age, years | 273 | 37 [34, 40] |
| Primiparous | 273 | 128 (46.9) |
| Conception through IVF-ET | 273 | 80 (29.3) |
| BMI at enrollment, kg/m^2^ | 273 | 28.5 [23.6, 32.0] |
| Prior HDP among multiparas | 145 | 88 (60.7) |
| Current smoking at enrollment | 273 | 12 (4.4) |
| Gestational age at initial visit, weeks | 273 | 9 [8, 11] |
| Gestational age at enrollment, weeks | 273 | 14 [12, 17] |
|  |  |  |
| Type of chronic hypertension |  |  |
| Newly diagnosed | 273 | 83 (30.4) |
| Pre-existing | 273 | 190 (69.6) |
|  |  |  |
| Antihypertensive medication at enrollment |  |  |
| No antihypertensive medication | 273 | 128 (46.9) |
| Methyldopa hydrate | 273 | 73 (26.7) |
| Calcium channel blocker | 273 | 50 (18.3) |
| Labetalol hydrochloride | 273 | 13 (4.8) |
| Others | 273 | 9 (3.3) |
|  |  |  |
| Aspirin initiation |  |  |
| ≤8 weeks | 273 | 18 (6.6) |
| ≤10 weeks | 273 | 44 (16.1) |
| ≤14 weeks | 273 | 134 (49.1) |
|  |  |  |
| Maximum systolic BP, mmHg |  |  |
| 8–9 weeks | 146 | 142.5 [131, 154] |
| 10–13 weeks | 262 | 141.5 [130, 151] |
| 14–18 weeks | 266 | 134 [126, 142] |
| At initial visit | 273 | 142 [131, 154] |
| At enrollment | 273 | 134 [126, 142] |
| Maximum diastolic BP, mmHg |  |  |
| 8–9 weeks | 146 | 91 [82, 99] |
| 10–13 weeks | 262 | 89 [82, 98] |
| 14–18 weeks | 266 | 84 [79, 91] |
| At initial visit | 273 | 91 [82, 99] |
| At enrollment | 273 | 85 [79, 91] |

Others included less frequently used antihypertensive agents such as hydralazine, doxazosin, azelnidipine, eplerenone, candesartan, and hydrochlorothiazide.

IQR: interquartile range; IVF-ET: *in vitro* fertilization and embryo transfer; BMI: body mass index; HDP: hypertensive disorders of pregnancy; BP: blood pressure

***Table S2. Participating Institutions and Collaborators***

***Institution***	***Location***	***Collaborator(s)***

Hokkaido University	Hokkaido	Ryo Tamaki, MD

Tenshi Hospital	Hokkaido	Aya Kawasaki, MD

Hirosaki University	Aomori	Maika Oishi, MD

Iwate Medical University	Iwate	Rie Oyama, MD

Tohoku University Hospital	Miyagi	Yusaku Kumagai, MD, PhD

Yamagata University	Yamagata	Norikazu Watanabe, MD

Takeda General Hospital	Fukushima	Shogo Kin, MD

Saitama Medical Center	Saitama	Yoko Takahashi, MD

Juntendo University Urayasu Hospital	Chiba	Akari Koizumi, MD

Kameda Medical Center	Chiba	Tokumasa Suemitsu, MD

The University of Tokyo	Tokyo	Mari Ichinose, MD

Kyorin University	Tokyo	Tomoko Shimada, MD

Nippon Medical School	Tokyo	Ikuno Kawabata, MD

Tokyo Metropolitan Bokutoh Hospital	Tokyo	Shinya Imada, MD

The Jikei University School of Medicine	Tokyo	Michiko Miya, MD

Tokyo Metropolitan Ohtsuka Hospital	Tokyo	Misako Iwata, MD

Kitasato University	Kanagawa	Yoshihiro Yoshimura, MD

Nippon Medical School Tama Nagayama	Tokyo	Eriko Kikuchi, MD

Hospital

Showa University	Tokyo	Shoko Hamada, MD

Tokai University	Kanagawa	Kanako Mitsuzuka, MD

Yokohama City University Medical Center	Kanagawa	Akiko Iwata, MD;
　　　　　　　　　　　　　　　　　　　　　　	Chika Akamatsu, MD

Yamato Municipal Hospital	Kanagawa	Aya Mochimaru, MD

Niigata University	Niigata	Kazufumi Haino, MD

Niigata City General Hospital	Niigata	Maomi Kamimura, MD

The University of Toyama	Toyama	Satoshi Yoneda, MD

Kanazawa University	Ishikawa	Kyosuke Kagami, MD

Fukui University	Fukui	Hiroshi Kawamura, MD

Shinshu University	Nagano	Yusuke Yokokawa, MD

Seirei Hamamatsu General Hospital	Shizuoka	Iga Kentaro, MD

Mie University	Mie	Masafumi Nii, MD

Shiga University of Medical Science	Shiga	Kaori Hayashi, MD

Japanese Red Cross Otsu Hospital	Shiga	Yasufumi Lee, MD

Kyoto University Hospital	Kyoto	Yoshitsugu Chigusa, MD

Kyoto Prefectural University of Medicine	Kyoto	Yukiko Tanaka, MD

National Hospital Organization Kyoto	Kyoto	Ikuko Emoto, MD
Medical Center

Mitsubishi Kyoto Hospital	Kyoto	Kyoko Nakatuma, MD

Osaka University	Osaka	Kazuya Mimura, MD

Osaka Women's and Children's Hospital	Osaka	Tetsu Wakimoto, MD

National Cerebral and Cardiovascular	Osaka	Atsushi Namanishi, MD
Center

Kitano Hospital	Osaka	Ayako Moribe, MD

Kindai University	Osaka	Yoshie Yo, MD

Kansai Medical University	Osaka	Aya Yoshida, MD

Kobe University	Hyogo	Hitomi Imafuku, MD

Kobe City Medical Center General Hospital	Hyogo	Saeko Tanabe, MD

Hyogo Prefectural Amagasaki General	Hyogo	Hiroshi Sato, MD
Medical Center

Nara Medical University	Nara	Ryuta Miyake, MD

Japanese Red Cross Wakayama Medical	Wakayama	Reiko Yokoyama, MD
Center

Tottori University	Tottori	Takashi Harada, MD

Okayama University Hospital	Okayama	Kazumasa Tani, MD

Kawasaki Medical School	Okayama	Mika Sugihara, MD

Kurashiki Central Hospital	Okayama	Hikaru Kiyokawa, MD, PhD

National Hospital Organization Kure	Hiroshima	Maiko Sagawa, MD;
Medical Center and Chugoku Cancer	Naofumi Yamane, MD

Center

Tsuchiya General Hospital	Hiroshima	Kaori Michikata, MD

Takamatsu Red Cross Hospital	Kagawa	Tatsuki Kadomoto, MD

Ehime University	Ehime	Yuka Uchikura, MD, PhD

Kyushu University Hospital	Fukuoka	Atsuhiko Sakai, MD

Kurume University	Fukuoka	Yusuke Kurokawa, MD

NHO Saga Hospital	Saga	Keisuke Tsumura, MD

Nagasaki University	Nagasaki	Noriko Miyashita, MD;
Noriko Shigetomi, MD

Kumamoto University Hospital	Kumamoto	Akihito Sagara, MD

Fukuda Hospital	Kumamoto	Tomoki Sato, MD;
Etsuko Saito, MD

University of Miyazaki	Miyazaki	Satoshi Matsuzawa, MD

Kagoshima City Hospital	Kagoshima	Hiroko Taniguchi, MD

University of the Ryukyus	Okinawa	Yukiko Chinen, MD

Okinawa Prefectural Chubu Hospital	Okinawa	Taisei Komatsu, MD

**Supplementary Figures**

***Figure S1. Study Timeline, Participant Flow, and Definition of the Primary Composite Outcome***

(A) Study timeline and periods of retrospective and prospective data collection.

(B) Participant flow diagram and components of the primary composite outcome.

BP, blood pressure; HDP, hypertensive disorders of pregnancy; HELLP, hemolysis, elevated liver enzymes, and low platelet count; SGA, small for gestational age; NICU, neonatal intensive care unit.

***Figure S2. Longitudinal Trends in Maximum Blood Pressure in the First Half of Pregnancy***

***Figure S3. Kaplan–Meier Estimates of Ongoing Pregnancy Without Composite Adverse Outcomes by Gestational Age***
